# 134. KRP-A218, an Orally Active and Selective PI4KB Inhibitor with Broad-Spectrum Anti-Rhinovirus Activity, Has Potent Therapeutic Antiviral Activity *In vivo*

**DOI:** 10.1093/ofid/ofab466.134

**Published:** 2021-12-04

**Authors:** Toshiyuki Matsui, Motomichi Fujita, Yuji Ishibashi, Tyzoon Nomanbhoy, Jonathan S Rosenblum, Michiaki Nagasawa

**Affiliations:** 1 Kyorin pharmaceutical Co., Ltd., Shimotsuga-gun, Nogi-machi, Tochigi, Japan; 2 Kyorin Pharmaceutical Co., Ltd., Shimotsuga-gun, Nogi-machi, Tochigi, Japan; 3 ActivX Biosciences, La Jolla, California

## Abstract

**Background:**

Rhinovirus (RV) is a major respiratory virus that poses a threat to immunocompromised people and those with underlying disease. However, there are no approved therapies. Moreover, RV infection cannot be prevented by a vaccine because there are over 100 serotypes. Here we report the pharmacological profile of a novel small-molecule host-targeted antiviral (HTA), KRP-A218 (A218). A highly potent and selective inhibitor of phosphatidylinositol 4 kinase beta (PI4KB), a key host factor of RV replication, A218 is undergoing clinical study.

**Methods:**

*in vitro* antiviral activities of A218 and Vapendavir (Vap), a virus-targeted antiviral, were examined by inhibition of CPE, viral load, or replication. *in vivo* antiviral activity and pathological analysis of A218 were examined in coxsackievirus B3 (CVB3; belong to the genus enterovirus as with RV)-infected mice as a surrogate model of RV infection as CVB3, unlike RV, replicates very well in both mouse and human tissue. Daily oral dosing of A218 (1-10 mg/kg) was started 2 days post intraperitoneal infection with CVB3. Tissue viral load, pancreas pathological change at 4 days post infection, and survival rate up to 14 days were evaluated. PI4KB heterozygous kinase-dead mice (PI4KB KD) were established by a CRISPR-Cas9 system. Viral load and survival rate following viral infection were evaluated in these mice.

**Results:**

A218 showed broad antiviral activity for RV and enteroviruses (Table) and has a higher barrier to drug resistance than Vap. These results are consistent with expectations for HTAs. Repeated dosing of A218 starting 2 days post infection decreased viral load and improved acute pancreatitis, accompanied by decrease of inflammatory and pancreatitis markers in plasma. Moreover, therapeutic dosing of A218 improved survival rate in a CVB3-infected lethal mouse model (Figure). These results show the first evidence that a PI4KB inhibitor has potent therapeutic efficacy in a severe viral infection model. Similar effects were observed in PI4KB KD, supporting the on-target effect of A218.

Table. Antiviral activity of A218 and Vap against RV/EV infection

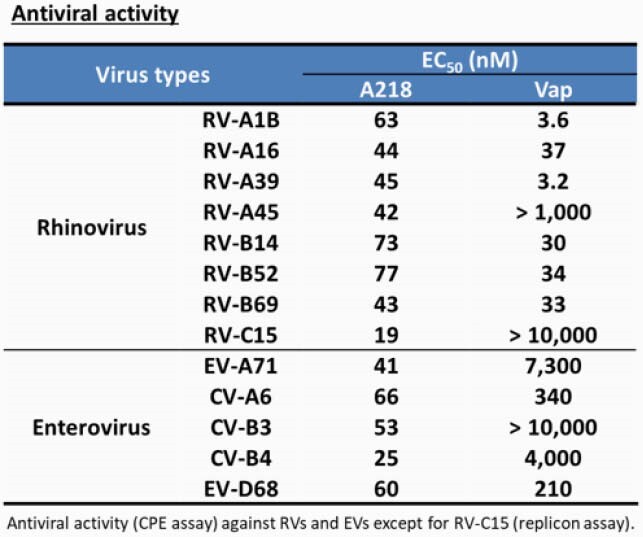

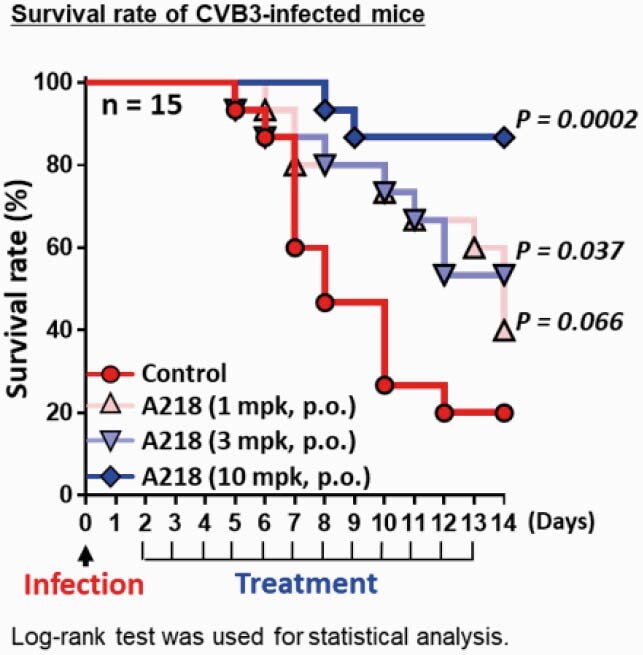

Figure. Therapeutic effect of A218 on survival rate in CVB3-infected mice

**Conclusion:**

A218 is a promising therapeutic agent for improving the exacerbation of pathological conditions caused by RV infection. Nonclinical package including GLP-Tox also supports the ongoing first-in-human study of A218.

**Disclosures:**

**Toshiyuki Matsui, MPharm**, **Kyorin pharmaceutical Co., LTD** (Employee) **Motomichi Fujita, PhD**, **Kyorin Pharmaceutical Co., Ltd.** (Employee) **Yuji Ishibashi, PhD**, **Kyorin Pharmaceutical co., ltd.** (Employee) **Tyzoon Nomanbhoy, PhD**, **ActivX Biosciences** (Other Financial or Material Support, Full time employee of ActivX, a wholly owned subsidiary of Kyorin Pharmaceuticals) **Jonathan S. Rosenblum, PhD**, **ActivX Biosciences** (Employee) **Michiaki Nagasawa, PhD**, **Kyorin Pharmaceutical Co., Ltd** (Employee)

